# Urbanization is associated with shifts in bumblebee body size, with cascading effects on pollination

**DOI:** 10.1111/eva.13087

**Published:** 2020-08-18

**Authors:** Panagiotis Theodorou, Lucie M. Baltz, Robert J. Paxton, Antonella Soro

**Affiliations:** ^1^ General Zoology Institute of Biology Martin Luther University Halle‐Wittenberg Halle (Saale) Germany; ^2^ German Centre for Integrative Biodiversity Research (iDiv) Halle‐Jena‐Leipzig Leipzig Germany

**Keywords:** *Bombus* spp., fragmentation, intertegular distance, land use, road density, temperature

## Abstract

Urbanization is a global phenomenon with major effects on species, the structure of community functional traits and ecological interactions. Body size is a key species trait linked to metabolism, life‐history and dispersal as well as a major determinant of ecological networks. Here, using a well‐replicated urban–rural sampling design in Central Europe, we investigate the direction of change of body size in response to urbanization in three common bumblebee species, *Bombus lapidarius*, *Bombus pascuorum* and *Bombus terrestris*, and potential knock‐on effects on pollination service provision. We found foragers of *B. terrestris* to be larger in cities and the body size of all species to be positively correlated with road density (albeit at different, species‐specific scales); these are expected consequences of habitat fragmentation resulting from urbanization. High ambient temperature at sampling was associated with both a small body size and an increase in variation of body size in all three species. At the community level, the community‐weighted mean body size and its variation increased with urbanization. Urbanization had an indirect positive effect on pollination services through its effects not only on flower visitation rate but also on community‐weighted mean body size and its variation. We discuss the eco‐evolutionary implications of the effect of urbanization on body size, and the relevance of these findings for the key ecosystem service of pollination.

## 1 INTRODUCTION

Of the human‐driven environmental changes, urbanization is arguably one of the most rapid and conspicuous. Urban land cover has increased considerably in the last decades (Seto, Güneralp, & Hutyra, 2012), with the proportion of the world's population residing in urban areas rising from 30% in 1950 to 55% in 2018 (United Nations, Department of Economic and Social Affairs, Population Division, 2019). Urban land cover is further forecast to increase by 0.6–1.3 million km^2^ by 2050, an expansion of 78%–171% of the global urban land area of 2015 (Huang, Li, Liu, & Seto, 2019).

The expansion of urban land cover is responsible not only for habitat loss, but also for several other environmental changes. These include the heat island phenomenon, pollution of air, water, light and noise (Grimm et al., 2008; McDonnell, Hahs, & Breuste, 2009), changes in habitat structure (i.e. an increase in impervious surfaces, habitat fragmentation and degradation) and changes in ecosystem processes such as nutrient cycling and primary productivity (Alberti, Correa, et al., 2017). As these rapid and drastic environmental changes constitute a challenge for organisms, urbanization can be a threat to biodiversity (McKinney, 2002; Moll et al., 2019). Furthermore, there is growing evidence that cities are important in contemporary evolution in that they accelerate phenotypic change in both animals and plants (Alberti, Correa, et al., 2017; Johnson & Munshi‐South, 2017; Rivkin et al., 2018; Seto et al., 2012).

Fragmentation is one of the most pervasive outcomes of urbanization. Fragments of vegetation within the built matrix of a typical cityscape, such as city parks, community gardens, private gardens and cemeteries, are usually small and isolated. This drastically affects the spatial distribution of resources, severely reduces the carrying capacity of every single fragment and limits its connectivity to other suitable fragments (Luck & Wu, 2002). The movement of organisms among fragments is therefore expected to be hampered at best, if not completely hindered. This can lead to severe constraints on the possibility of single individuals within a population to find suitable mating partners or enough resources to sustain themselves. It can also lead to negative consequences on the genetic diversity of populations within fragments and increased genetic differentiation among fragments (Cote et al., 2017; Johnson & Munshi‐South, 2017). Limited dispersal capabilities in a fragmented landscape make populations more susceptible to inbreeding and the deleterious effects of genetic drift (Bohonak, 1999). Dispersal is indeed a key ecological process for avoidance of kin competition (Hamilton & May, 1977), regulation of population density (Clobert, Baguette, & Benton, 2012), the maintenance of genetic diversity (Clobert et al., 2012) and colonization of new areas (Cote et al., 2017; Duputié & Massol, 2013). Traits related to dispersal capability are therefore likely to be exposed to strong selection and expected to be the prime target for evolutionary change under fragmentation (Cheptou, Hargreaves, Bonte, & Jacquemyn, 2017).

Among the morphological traits related to dispersal, body size has received particular attention; it is generally an important predictor of dispersal for large and diverse taxonomic groups, including butterflies, birds and mammals (Ottaviani, Cairns, Oliverio, & Boitani, 2006; Stevens et al., 2013; Whitmee & Orme, 2013), but not universally for all taxa (see Merckx, Kaiser, & Van Dyck, 2018). Body size is a continuous trait, intrinsically linked to metabolism and intricately tied to important functions such as growth, survival and reproduction (Horne Curtis, Hirst Andrew, & Atkinson, 2017). It is critical for its effects on individual longevity, fecundity, the ability to migrate, competitive, predatory and antipredatory abilities, and on the ability of organisms to withstand starvation and desiccation (Atkinson, 1994). As such, body size is expected to be optimized by natural selection, even if within obvious phylogenetic constraints. Therefore, if the selective regime of urban settings is one imposed by low connectivity of ecological resources, and greater body size has a mitigating effect because of the enhanced dispersal capability it confers, then body size is expected to increase in response to the anthropogenic fragmentation brought on by urbanization. Actively dispersing arthropods show increased investment in flight muscle mass (Merckx & Van Dyck, 2006; Thomas, Hill, & Lewis, 1998), longer legs (San Martin y Gomez & Van Dick, 2012) and increased body size at population and community levels (Merckx, Kaiser, et al., 2018; Piano et al., 2017) in response to fragmentation. This trend was recently supported by a wide‐ranging study (Merckx, Souffreau, et al., 2018), which showed that communities of taxa (aquatic and terrestrial) with a positive size‐dispersal link had shifted to larger body sizes along a gradient of increased urbanization (Piano et al., 2017).

Bees are a taxonomic group with a positive association between body size and flight capability (Greenleaf, Williams, Winfree, & Kremen, 2007) that are negatively affected by habitat loss and fragmentation (Winfree, Aguilar, Vásquez, LeBuhn, & Aizen, 2009). Moreover, body size in bees is a significant predictor of genetic differentiation, with larger bees exhibiting less differentiation (López‐Uribe, Jha, & Soro, 2019). This is further evidence that larger bees might be able to disperse more, if population genetic structure is taken as an indirect measure of gene flow (Bohonak, 1999; Broquet & Petit, 2009).

Bumblebees (genus *Bombus*) are a major group of bees comprising approximately 250 species worldwide that vary greatly in coloration, tongue length, nesting biology, habitat use and body size (Goulson, 2010). They are social, with annual colonies founded by a queen that is typically singly mated (Schmid‐Hempel & Schmid‐Hempel, 2000). They are fairly large (ranging from 1 cm up to 4 cm long) compared to other bee species, covered in dense pile, capable of endothermy and thus well adapted to cool conditions (Goulson, 2010). Most bumblebee species are generalist pollinators and, as such, facilitate the reproduction of a large number of wild plants and commercial crops in temperate regions (Goulson, 2010). They are also the best‐studied group of non‐*Apis* bees and have become a model system for investigations of behaviour, ecology and evolution, in part because of their ecological and economic importance (Woodard et al., 2015). Like all bees, bumblebees require suitable habitat for foraging, nesting and for queen overwintering. Habitat fragmentation and loss of foraging habitat have therefore a negative impact on bumblebee survival (Carvell et al., 2017; Goulson, Lye, & Darvill, 2008), while food availability is positively associated with the production of gynes and/or males (Crone & Williams, 2016; Rundlöf, Persson, Smith, & Bommarco, 2014). Not surprisingly, the distribution and abundance of floral resources affect foraging distances, which increase where resources are sparse and scattered (Jha & Kremen, 2013; Pope & Jha, 2018; Westphal, Steffan‐Dewenter, & Tscharntke, 2006). Importantly, phenotypic changes in bumblebees along environmental gradients are apparent for a number of traits; in North America, bumblebee worker body size has decreased across 125 years (Nooten & Rehan, 2019); also in North America, two alpine bumblebee species responded to a decline in floral resources by evolving shorter tongues (Miller‐Struttmann et al., 2015). Finally, in Switzerland, even if in contradiction to our outgoing hypothesis, mean body size, proboscis length, wing length and corbicula length were all shown to be smaller in urban versus rural bumblebee populations (Eggenberger et al., 2019). Interestingly, signatures of selection associated with urbanization have been found recently in the bumblebee species *B. lapidarius* (Theodorou et al., 2018), suggesting potential adaptation to urban environments.

As a taxon with a positive association between body size and dispersal (López‐Uribe et al., 2019) and foraging capabilities (Greenleaf et al., 2007), bumblebees are expected to show a positive body size shift in response to fragmentation (Merckx, Souffreau, et al., 2018). Fragmentation has indeed been proposed as the factor explaining an increase in queen bumblebee body size over the last century in Belgium (Gérard et al., 2019).

Nevertheless, cities are not only more fragmented, they are also generally warmer than surrounding rural areas because of the heat island effect (Manoli et al., 2019). Large bees are known to be more vulnerable to overheating in hot ambient air temperatures compared to small individuals because of their lower ratio of surface area to volume (Goulson, 2010). Thus, if temperature were the main factor affecting bee performance, smaller body sizes would be expected in cities. Indeed, Eggenberger et al. (2019) attributed the observed decline in mean bumblebee body size in Swiss cities to urban warming and local availability of floral resources, not urban fragmentation, even though the study lacked information on habitat characteristics.

Body size is a key trait not just because it determines home range size and dispersal ability and thus how species respond to fragmentation, but also because it affects how species interact. For example, body size influences the structure and dynamics of ecological networks, including plant–pollinator interactions (Hagen et al., 2012; Woodward et al., 2005). Large bumblebee foragers have greater visual acuity, larger brains and greater antennal sensitivity, they are better in learning and memory, are less preyed upon, visit more flowers per unit time and are capable of depositing a higher number of pollen grains on stigmas compared to smaller foragers (Goulson et al., 2002; Mares, Ash, & Gronenberg, 2005; Spaethe & Weidenmüller, 2002; Stout, 2000; Willmer & Finlayson, 2014; Worden, Skemp, & Papaj, 2005). Therefore, intraspecific body size variation in bees could influence the ecosystem service of pollination via its effects on foraging behaviour and the efficiency of pollen transfer.

Here, we analysed the direction of change and the main environmental correlates of body size in three bumblebee species using a paired design across 18 rural and urban landscapes in Central Europe. We used two bumblebee species, *B. lapidarius* and *B. pascuorum*, which are known to forage close to their nests, and the long distance forager, *B. terrestris* (Knight et al., 2005). To minimize the effects of local floral resource availability, we sampled bumblebees from flowering plant‐rich sites in rural and urban ecosystems. In order to investigate whether body size shifts are potentially important in influencing bumblebee performance as ecosystem function providers, we also correlated bumblebee body size with seed set of potted, bumblebee‐pollinated *Trifolium pratense* experimental plants. Our two main objectives were as follows: (a) to assess whether there is a population‐level response of bumblebee body size to urbanization and its potential causes (body size as “response trait” Lavorel & Garnier, 2002); (b) to evaluate the community level impact of the response to urbanization of body size for the ecosystem service of pollination (body size as “effect trait” Lavorel & Garnier, 2002).

If the selective regime in urban settings is the one imposed by fragmentation of ecological resources and if greater body size has a mitigating effect because of the increased bridging potential it confers, we expect bumblebee body size to be larger in urban environments, with potential enhancement of the ecosystem service of pollination. In the contrary, if the selective regime in cities is one imposed by urban warming, we expect bumblebee body size to be smaller in cities with a consequential reduction in pollination service provision.

## 2 MATERIALS AND METHODS

### 2.1 Study species

For our study, we used the buff‐tailed bumblebee *Bombus terrestris* (Linnaeus 1758), the red‐tailed bumblebee *Bombus lapidarius* (Linnaeus 1758) and the common carder bee *Bombus pascuorum* (Scopoli 1763), all common and widespread in Europe (Goulson, 2010) and among the most abundant bee species in Central Germany (Theodorou et al., 2016, 2020). All species can be found across a range of semi‐natural and managed habitats, including urban ones (Goulson, 2010; Polce et al., 2018; Theodorou et al., 2016), and thus offer an excellent model system to study how land use can affect bee communities and species functional traits. *Bombus lapidarius* and *B. pascuorum* forage over similar ranges (minimum estimated maximum range: 450 m, (Knight et al., 2005)), while *B. terrestris* forages over a greater range (minimum estimated maximum range: 758 m, (Knight et al., 2005)). *Bombus terrestris* and *B. lapidarius* nest in subterranean holes whereas *B. pascuorum* generally constructs nests on or close to the soil surface in dense vegetation or leaf litter (Goulson, 2010).

### 2.2 Study design and sampling

We collected *B. terrestris*, *B. lapidarius* and *B. pascuorum* workers in June–August 2014 from 18 flowering plant‐rich sites located in nine German cities (Berlin, Braunschweig, Chemnitz, Dresden, Göttingen, Halle, Jena, Leipzig and Potsdam) and in nine corresponding (i.e. paired) rural locations (Figure S1). All our urban sites were botanical gardens and parks located within the urban core of each city, surrounded by a high density of roads and human infrastructure (Table S1). Rural sites were selected using Quantum GIS (QGIS.org, 2020) and land‐use maps to be dominated by agricultural land and semi‐natural/forest cover, typical of the region's rural environment. To select rural sites, we drew a buffer of at least 10 km from an urban site and then used Quantum GIS to identify areas with semi‐natural vegetation, that were largely devoid of “residential” cover, had a low density of roads and were dominated by arable land and semi‐natural/forest cover within the surrounding 1 km radius (Table S2). To ensure independence of sampling, we selected sites at least 10 km distance within each pair (Figure S1), which is beyond the foraging distances of the *Bombus* species used in our study (Goulson, 2010).

Using hand‐nets, we collected as many foragers as possible of the three species. Within a period of two days at each site (i.e. with identical sampling effort across species and sites), we collected 672 *B. terrestris* workers (range: 21–59 individuals per site), 438 *B. lapidarius* workers (range: 6–52 individuals per site) and 721 *B. pascuorum* workers (range: 37–54 individuals per site), within 250 m of the centre of our selected flowering plant‐rich sites. Sampling was conducted during hours of high bumblebee activity (0900–1700). Temperatures exceeded 16°C, wind speed was less than 2 m/s at 1 m above ground level, and skies were sunny on all sampling days (Table S3). Individuals were kept in 95% ethanol and stored at −20°C.

Due to the difficulty in distinguishing workers of *B. terrestris* from those of *Bombus lucorum* (Linnaeus 1761), a similar species in morphology and ecology, we used DNA barcoding (DNA sequences of the “barcode” fragment of the mitochondrial *cytochrome oxidase I* gene) of a random subset of individuals (17 individuals from each site) to evaluate the commonness of each species in our samples. Our analyses revealed that *B. lucorum* was rare (9.5% of all collected specimens). The proportion of detected *B. lucorum* individuals did not differ between the two ecosystems (8.75% in urban and 9.55% in rural; LMM (linear mixed model), *t* = 1.69, *p* = .276). Due to the rarity of *B. lucorum* in our samples, its ecological and body size similarity with *B. terrestris* (intertegular distance: *B. lucorum*, x̅ = 3.82 ± 0.35 *SD*; *B. terrestris,* x̅ = 3.85 ± 0.42 *SD*; LMM; χ^2^ = 0.595, *p* = .440), all nonbarcoded specimens were combined as *B. terrestris* in our downstream analyses. In principle, we cannot exclude that some of the *B. terrestris* individuals we sampled derived from commercial hives, especially in the rural sites, where they are more likely to be employed in greenhouse crop pollination. Nevertheless, there were no greenhouses in the vicinity of our sampling locations. Moreover, to the best of our knowledge, there is no evidence that commercial *B. terrestris* are systematically different in size from wild conspecifics. Thus, even if we had collected some commercial bumblebees, their negligible number is very unlikely to have affected our results. That the shift in body size that we observed (see results) was detected in all three species and not just *B. terrestris* further suggests that patterns were not biased by commercial *B. terrestris*.

### 2.3 Body size measurements

As a proxy for body size, we used intertegular distance (ITD), the distance between the two insertion points of the wings (tegulae). ITD is a standard measure of body size in bumblebees that is highly correlated with dry body mass (Cane, 1987). It serves as an indicator for the volume of the thoracic flight musculature (Cane, 1987), is strongly correlated with species mobility (Greenleaf et al., 2007) and it is therefore considered an indication of dispersal ability. ITD was measured using a stereo microscope (Olympus SZX7) with an integrated camera and the digital measurement tool in the cellSens software v.1.6. We additionally calculated the percentage coefficient of variation (CV = 100 • *σ*/*μ*, where *σ* is the standard deviation and *μ* is the mean value) of ITD.

### 2.4 Ecosystem service of pollination

At each site, we evaluated the pollination success of red clover, *Trifolium pratense* (Linnaeus 1753), a self‐incompatible plant species with papilionaceous flowers that is preferentially visited by bumblebees (Goulson, 2010; Theodorou et al., 2016, 2017, 2020). Seeds of *T. pratense* were obtained from a local seed provider (Rieger‐Hofmann GmbH, Blaufelden, Germany) and were germinated and grown for two months in an insect‐free glasshouse before placement at our study sites. Ten potted plants each with eight open inflorescences marked with coloured tape were placed at each field site for 5 days during bumblebee sampling dates, with each pair of sites (urban and rural pair) sampled on the same five days. To evaluate the dependence of *T. pratense* on insect visitation for seed set, one inflorescence was bagged in each plant to prevent visitation by pollinators. Bagged inflorescences did not set any seed, demonstrating the dependence of *T. pratense* on insect visitation for seed set. Plants in each site were randomly ordered at one metre distance along a transect of 10 m × 1 m.

At each site, we monitored all flying insects visiting the experimental plants in order to identify their main flower visitors. Individual plants were observed twice per site per sampling day (15 min in the morning and 15 min in the afternoon), for a total of 300 min observation time of *T. pratense* per site. Visitor identity (11 morphogroups: 1. Coleoptera; 2. Syrphidae; 3. other Diptera; 4. Lepidoptera; 5. wasps; bees (6. Andrenidae, 7. Halictidae, 8. *Bombus lapidarius*, 9. *Bombus terrestris,* 10. *Bombus pascuorum* and 11. *Apis mellifera*)) was recorded. Furthermore, at each site we estimated the abundance of conspecific pollen donors by counting the number of inflorescences of co‐flowering *T. pratense* plants within a 200 m buffer around each plot (Table S4).

When the five days of the pollination experiment were completed at a site, focal plants were returned to the insect‐free greenhouse until seeds were formed. Seeds from all seven unbagged inflorescences per plant were counted and the average number of seeds per plant (i.e. per 7 inflorescences) was used as a measure of the ecosystem service of pollination.

### 2.5 Environmental variables

To determine the main environmental correlates of body size in both rural and urban flower‐rich sites, we gathered a series of environmental variables. We quantified local flowering plant abundance at each site as an estimator of floral resource availability using 10 randomly placed 1 m^2^ quadrats at each site (Table S4). As we sampled at each site, we also used a weather metre (Kestrel 4000; Nielsen‐Kellerman) to measure wind speed and temperature 1 m above ground level. Average wind speed and temperature during morning (between 0930–1000 am) sampling were used as explanatory variables in our downstream analyses. Roads fragment a landscape, affect bumblebee densities and act as barriers to movement (Bhattacharya, Primack, & Gerwein, 2003; Kallioniemi et al., 2017). Thus, using Quantum GIS (QGIS.org, 2020) with data obtained from Geofabrik GmbH we quantified road density at multiple spatial scales (250, 500, 750 and 1,000 m) around the centre of each site (quantified as the total length of road per site at that scale) as a metric of fragmentation. To identify the scale at which road density had the most power to explain body size variation in each bumblebee species, we correlated each species’ ITD with road density at each of our study sites at all four scales. Correlation coefficients peaked at the 250 m scale for *B. lapidarius* and *B. pascuorum* and at the 1,000 m scale for *B. terrestris* (Table S5). These scales were used for subsequent analyses.

Ecotones (transitions between two habitat types) and green cover could be important habitats that provide resources for bumblebees (Theodorou et al., 2020). We therefore also quantified the proportion of green cover (semi‐natural and forest cover, botanical gardens, public parks and allotments) and edge density (ecotones), as total length of “green cover” of edge patches (semi‐natural and forest cover, botanical gardens, public parks and allotments) divided by their total area, which represents a quantification of landscape configuration. To do so, we used Quantum GIS with data obtained from Geofabrik GmbH. Similarly to road density, we quantified the proportion of green cover and edge density at multiple spatial scales (250, 500, 750 and 1,000 m) and identified the most appropriate scale for downstream analyses by correlating each species’ ITD with the proportion of green cover and edge density at each of our study sites at all four scales. Correlation coefficients peaked at the 1,000 m scale for all bumblebee species for both the proportion of green cover and edge density (Tables S6 and S7).

### 2.6 Statistical analyses

Prior to each analysis, we standardized each predictor (mean of zero and standard deviation of one) and we used variance inflation factors (VIFs) with a cut‐off of five to check for collinearity among our explanatory variables (Zuur, Ieno, Walker, Saveliev, & Smith, 2009). As *road density* and *ecosystem type* (rural/urban) were highly collinear in all our models, we conducted separated analyses with each of the variables while excluding the other.

To test for differences between urban and rural sites in their road density at 250 m and 1,000 m scales, total green cover at the 1,000 m scale, edge density at the 1,000 m scale, wind speed and ambient temperature at sampling, we used linear mixed models (LMMs) with site pair as a random effect factor. We used generalized linear mixed models (GLMMs) with a negative binomial error structure to test for differences between urban and rural sites with respect to bumblebee and honeybee visitation rates to our *T. pratense* experimental plants. Again, site pair was included as a random effect factor.

We tested for differences in bumblebee body size and variation in body size between rural and urban sites using LMMs with site pair as a random effect factor. Ecosystem type (rural/urban) and its interaction with bumblebee species identity, day of the year, temperature when sampling, wind speed, flowering plant abundance, edge density, proportion of green cover and honeybee visitation rates were used as fixed factors. When investigating the main drivers of body size variation, we also used bumblebee sample sizes as a covariate. In two separate models, we replaced *ecosystem type* with *road density* at either 250 or 1,000 m scales.

To investigate the main correlates of *T. pratense* seed set, we used LMMs with site pair as a random effect factor. Ecosystem (rural/urban), insect visitation rates to our experimental plants, community‐weighted mean (CWM) of bumblebee body size, community‐weighted mean coefficient of variation (CWM CV) of bumblebee body size, local flowering plant abundance and conspecific pollen donor availability were used as fixed factors. We used CWM because it is a trait‐based index known to be one of the best predictors of ecosystem functioning (Gagic et al., 2015; Woodcock et al., 2019). We calculated the CWM of ITD and the CWM CV of ITD using the R packages “tidyr” and “dplyr”(Wickham, François, Henry, & Müller, 2019).

All mixed model analyses were performed using the R package “lme4” (Bates, Mächler, Bolker, & Walker, 2015). For all models, we performed model selection to determine the most parsimonious model using the *step* function (backward elimination) within the R package “lmerTest” (Kuznetsova, Brockhoff, & Christensen, 2017). The P‐values for the fixed effects are calculated from *F* tests based on Sattethwaite's or Kenward–Roger approximation (Kuznetsova et al., 2017). Significance of differences in body size between bumblebee species was tested with Tukey's HSD post hoc method using the R package “multcomp” (Hothorn, Bretz, & Westfall, 2008). All model assumptions were checked visually and were found to conform to expectations (residuals normally distributed, homogeneity of variance, linearity, Supplementary Figure Model Assumptions).

In addition to our multiple regressions models, we performed piecewise structural equation modelling (*SEM*) to evaluate causal relationships between environmental variables, bumblebees and red clover pollination. *SEM* it is a framework for quantitative analysis that enable the inference of causal relationships between variables of interest. It starts with the construction of a model, which encodes a set of assumptions (derived from prior knowledge) of causal relationships between those variables, and is typically represented by a path diagram of boxes (representing measured variables), connected by unidirectional arrows (links), which are explicit hypotheses of causal relationships (Pearl, 2012). We hypothesized that ecosystem, temperature at sampling, wind, local resource availability, conspecific pollen donor availability, edge density, proportion of green cover and day of the year might have affected red clover seed set directly or indirectly through affecting visitation rates, bumblebee CWM body size and CWM CV of body size. We performed piecewise *SEM* analyses using the R package “piecewiseSEM” (Lefcheck, 2016), separately for *road density* and *ecosystem type*. We used the d‐separation (d‐sep) test to evaluate whether the models could be improved with the exclusion of hypothesized path(s) or inclusion of any nonhypothesized independent path(s), within the set of included variables. We used Fisher's C statistic for evaluating the fit of our piecewise *SEM* (Shipley, 2009). Path coefficients and deviance explained were then calculated for each model. We report both conditional (*R* ^2^ *_c_*, all factors) and marginal (*R* ^2^ *_m_*, fixed factors only) coefficients of determination for the linear mixed effect models incorporated in the *SEM* (Nakagawa & Schielzeth, 2013). We used Sobel's method to test for significant indirect effects (Sobel, 1982).

All statistical analyses were performed in R v. 3.5.2 (R Core & Team, 2018).

## 3 RESULTS

A total of *n* = 1829 *Bombus* workers were measured, *n* = 931 from urban sites (*B. terrestris,* *n* = 337; *B. lapidarius,* *n* = 201 and *B. pascuorum,* *n* = 393; Figure S2) and *n* = 898 from rural sites (*B. terrestris,* *n* = 333; *B. lapidarius,* *n* = 237 and *B. pascuorum,* *n* = 328 Figure S2). The number of collected individuals did not differ between urban and rural ecosystems (GLMM; *B. terrestris*, χ^2^ = 0.013, *p* = .909; *B. lapidarius*, χ^2^ = 0.515, *p* = .472; *B. pascuorum*, χ^2^ = 0.751, *p* = .386; Figure S2). Intertegular distance ranged from 2.66 to 5.90 mm for *B. terrestris* (*n* = 672, x̅ = 3.85 ± 0.42 *SD*), from 2.29 to 4.38 mm for *B. lapidarius* (*n* = 672, x̅ = 3.54 ± 0.31 *SD*) and from 2.01 to 4.26 mm for *B. pascuorum* (*n* = 721, x̅ = 3.31 ± 0.35 *SD*).

Road density was higher in urban compared to rural ecosystems (LMM; 250 m scale; χ^2^ = 38.78, *p* < .001 (Rural: 1.063 ± 0.483 *SD*; Urban: 4.934 ± 1.740 *SD*); 1,000 m scale; χ^2^ = 70.43, *p* < .001 (Rural: 17.476 ± 5.444 *SD*; Urban: 82.176 ± 20.556 *SD*); Figure S3). However, total green cover, edge density, wind speed and temperature at sampling did not differ between ecosystems (LMM, χ^2^ = 0.006, *p* = .936; χ^2^ = 0.237, *p* = .626; χ^2^ = 2.469, *p* = .116; χ^2^ = 0.013, *p* = .907, respectively, Figure S3). Furthermore, no significant correlation was found between day of the year of sampling and ambient temperature when sampling (across both ecosystems: *r* = .272, *p* = .273; within urban sites: *r* = .303, *p* = .427; within rural sites: *r* = .260, *p* = .497), probably because we chose to sample bees on fine, sunny days in summer.

### 3.1 Main environmental correlates of intraspecific bumblebee body size

**Figure 1:**
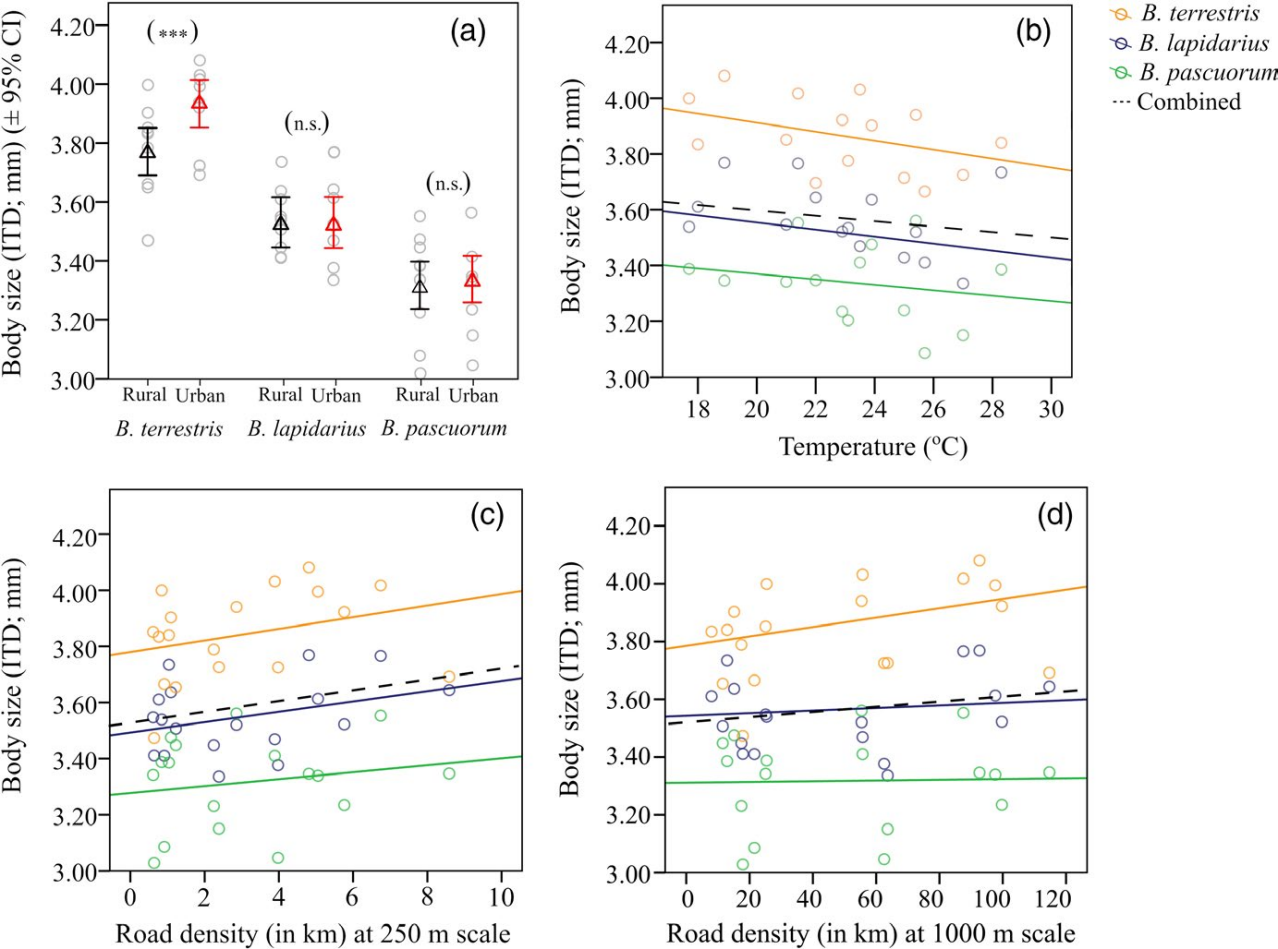
(a) Mean body size (± 95% CI) per species across all rural (black) and all urban (red) sites (n.s., not significant; ***p < .001). Relationships between *Bombus* body size and (b) ambient temperature when sampling, (c) road density at the 250 m scale and (d) road density at the 1,000 m scale, both as length of roads within a site for that scale. Plotted lines show predicted relationships

Overall, we found evidence of an interaction between ecosystem type and species identity on body size, suggesting that any pattern of body size shift between ecosystems is not consistent across *Bombus* spp. (Table 1 (model 1), Figure 1a). Indeed, the mean body size of *B. lapidarius* and *B. pascuorum* did not differ between ecosystems (Tukey HSD; *Z* = −0.073, *p* = .942, Z = 0.621, *p* = .535, respectively; Figure 1a). In contrast, *B. terrestris* individuals were significantly larger in cities (Tukey HSD; *Z* = 4.913, *p* < .001; Figure 1a). In addition, temperature when sampling (Table 1 (model 1); Figure 1b) and day of the year (Table 1 (model 1); Figure S4) were important predictors of bumblebee body size across ecosystems; body size dropped with increasing ambient temperature and with the progression of summer. When replacing *ecosystem (rural/urban)* with *road density* at the 250 m scale, our best LMM revealed that body size of all three *Bombus* spp. was larger in sites associated with high road density (Table 1 (model 2); Figure 1c). When replacing *ecosystem (rural/urban)* with *road density* at the 1,000 m scale, only *B. terrestris* body size was larger in sites associated with high road density ((Table 1 (model 3); LMM; *t* = 2.112, *p* = .034; Figure 1d), indicating that the body size response to road density is species and scale dependent. Models did not include wind speed, local floral resource abundance, edge density, proportion of green cover and honeybee visitation rates.

**Table 1:** Best linear mixed effect models explaining body size (ITD; mm), body size variation (coefficient of variation (CV) of ITD as %) and *Trifolium pratense* seed set across all our sampling sites. Model 1 differs from models 2 and 3 in that the predictor *ecosystem type* of model 1 is replaced by *road density* in models 2 and 3 (respectively at the 250 m and 1,000 m scale). See Table S8 for beta coefficients and related statistics

Response variable	Predictors	*F*‐value	*df*	*p*‐value
Body size (model 1)	Temperature	11.653	1	<.001***
	Day of the year	7.049	1	.032*
	Ecosystem type: Species identity	6.684	2	.001***
Body size (model 2)	Temperature	9.017	1	.002**
	Day of the year	8.718	1	.020*
	Species identity	372.651	2	<.001***
	Road density (250 m)	9.051	1	.002**
Body size (model 3)	Temperature	8.725	1	.003**
	Day of the year	7.901	1	.025*
	Road density (1,000 m): Species identity	5.185	2	.005**
CV body size (model 4)	Species identity	4.409	2	.017*
	Temperature	11.373	1	.001***
	Sample size	0.944	1	.336
*T. pratense* seed set (model 5)	Visitation rates	23.045	1	<.001***
	CWM body size	7.906	1	.006**
	CWM of CV body size	5.510	1	.021*

Abbreviation:

*df*, degrees of freedom.

* *p* ≤ .05;

** *p* ≤ .01;

*** *p* ≤ .001.

### 3.2 Main environmental correlates of intraspecific coefficient of variation in bumblebee body size

**Figure 2:**
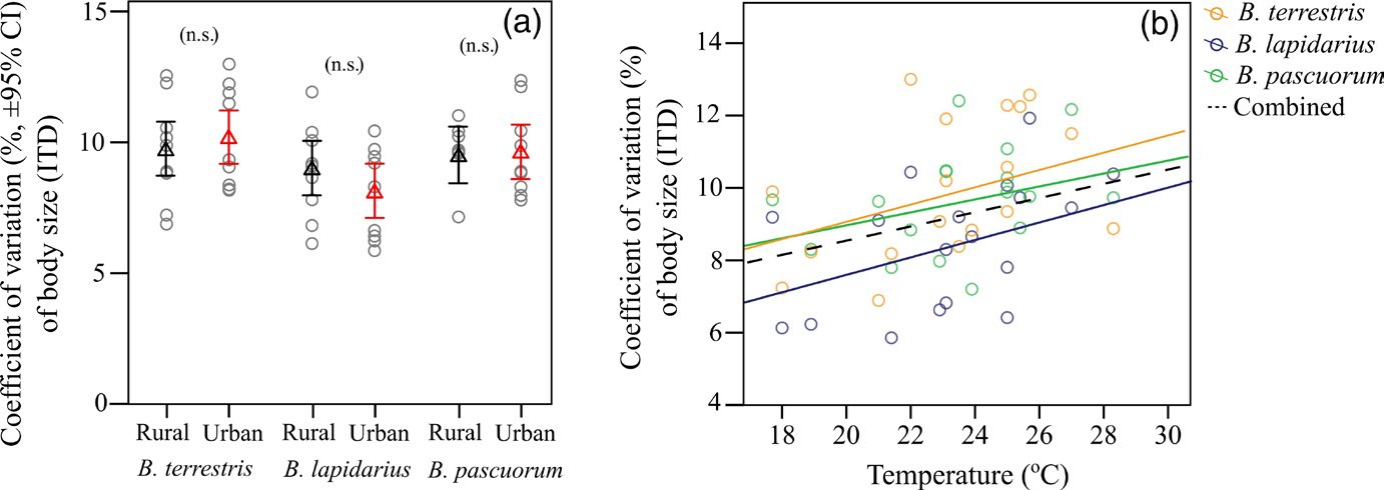
(a) Mean body size variation (± 95% CI) per *Bombus* species across all rural (black) and all urban (red) sites (n.s., not significant). (b) Relationship between *Bombus* body size coefficient of variation (%) and ambient temperature when sampling. Plotted lines show predicted relationships

*Bombus* species identity and temperature were the most important predictors of variation in body size (Table 1 (model 4); Figure 2). *Bombus terrestris* exhibited greater variation in body size compared to *B. lapidarius* (Tukey HSD; *Z* = 2.798, *p* = .005). Body size variation did not differ either between *B. terrestris* and *B. pascuorum* (Tukey HSD; *Z* = 0.816, *p* = .414) or between *B. pascuorum* and *B. lapidarius* (Tukey HSD; *Z* = 1.815, *p* = .065). Ecosystem type or road density, day of the year, wind speed, local floral resource abundance, edge density, proportion of green cover and honeybee visitation rates were not included in the most parsimonious model.

### 3.3 Bumblebee community‐weighted body size and pollination service provision

Bumblebees were the dominant flower visitors of red clover across all sites. During the 5,400 min of direct observations of our *T. pratense* experimental plants, we observed a total of 1,306 interactions between flying insects and red clover flowers across our 18 sites. Bumblebees were involved in 75.3% (*n* = 984), *Apis mellifera* in 8.7% (*n* = 114), halictid bees in 5.1% (*n* = 65), Lepidoptera in 4.6% (*n* = 60), andrenid bees in 2.4% (*n* = 31), syrphid flies in 2.1% (*n* = 27), other Diptera in 1.3% (*n* = 17) and Coleoptera in 0.5% (*n* = 6) of these interactions. We found higher bumblebee and honeybee visitation rates in urban compared to rural sites (Bumblebees: Rural = 2.988 ± 2.372 *SD*; Urban = 7.944 ± 6.038 *SD*, GLMM; χ^2^ = 6.985, *p* = .006; Honeybees: Rural = 0.266 ± 0.790 *SD*; Urban = 1.000 ± 1.593 *SD*; GLMM; χ^2^ = 8.495, *p* = .003).

**Figure 3:**
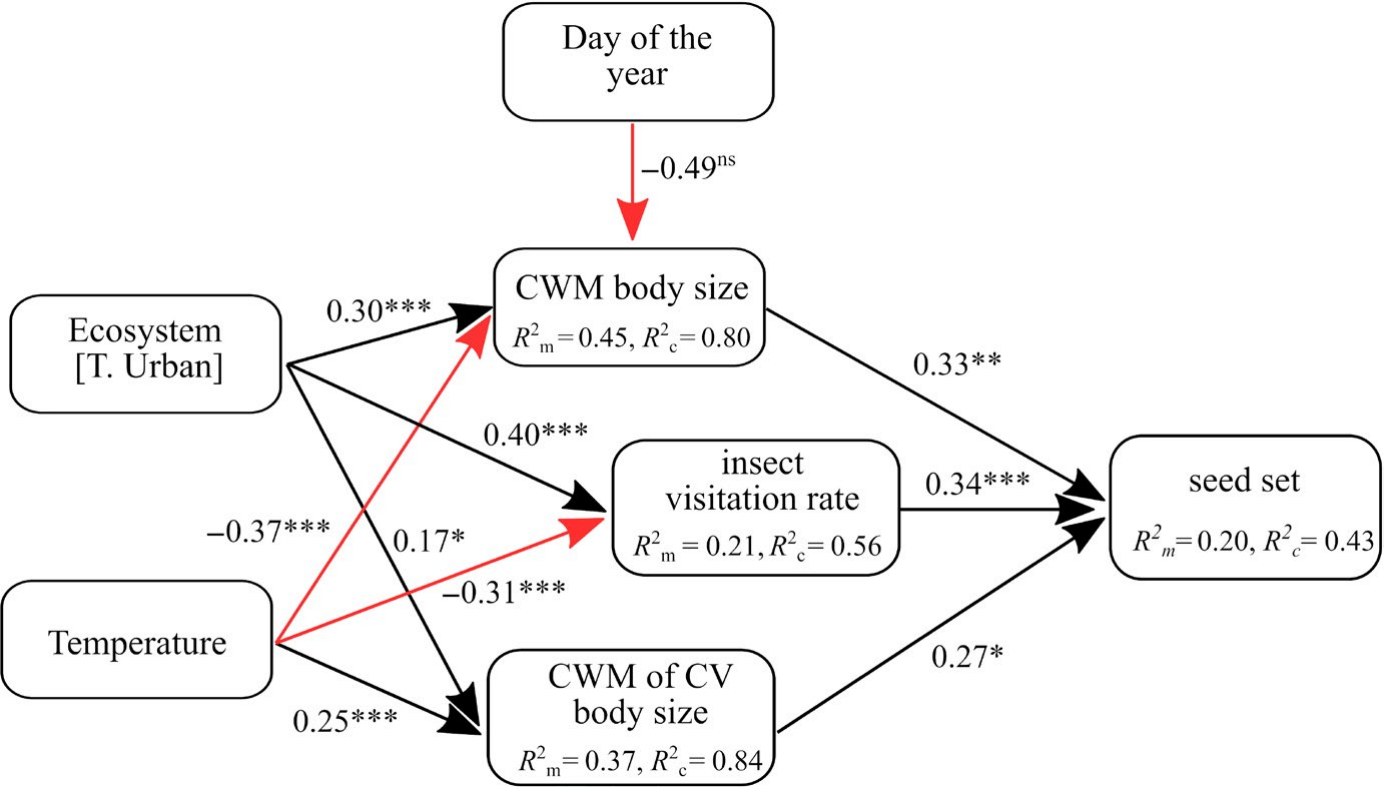
Representation of the structural equation model of urbanization and ambient temperature when sampling, their relationships with bumblebee community‐weighted mean (CWM) body size and CWM of the coefficient of variation (CV) of body size, and the effects of visitation rates and body size on pollination. T. Urban, urban treatment of this categorical variable. Black solid arrows show positive and red arrows negative effects, as derived from the piecewise *SEM* analysis. Standardized path coefficients are reported next to the bold arrows and *R* ^2^ values (conditional *R* ^2^ *_c_* and marginal *R* ^2^ *_m_*) are reported for all response variables. ns not significant; *p ≤ .05; **p ≤ .01; ***p ≤ .001

**Figure 4:**
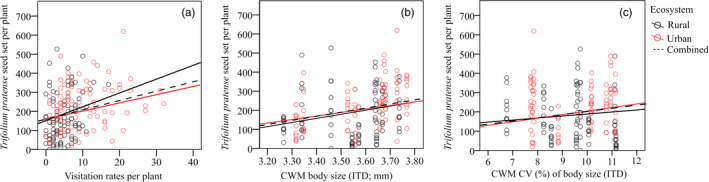
Relationships between *Trifolium pratense* seed set per plant and (a) visitation rates of all visitors per 30 min per plant; (b) CWM *Bombus* spp. body size (in mm) and (c) CWM CV of *Bombus* spp. body size (%). Plotted lines show predicted relationships

Our piecewise *SEM* selection process yielded one final path model relating red clover seed set with community‐weighted body size and environmental variables, with stable fit to our data (Fisher's C = 15.253, *df*=12, *p* = .228; Figure 3). We found a significant effect of urbanization on CWM body size, CWM CV of body size and visitation rates (*p* < .05; Figure 3; Table S9). We also found a positive effect of ambient temperature at sampling on the CWM of body size variation and a negative effect of ambient temperature on CWM of body size and visitation rates (*p* < .001; Figure 3; Table S9). Visitation rates, CWM of body size and CWM of body size variation were important positive predictors of red clover seed set (*p* < .05; Table 1 (model 5), Figures 3 and 4; Table S9). We found a negative indirect effect of ambient temperature on red clover seed set mediated by CWM of body size and visitation rates (Sobel test; −2.317, *p < *.05). We found a positive indirect effect of urbanization on red clover seed set mediated by CWM of body size, CWM of body size variation and visitation rates (Sobel test; 3.566, *p < *.001). Separate SEMs, in which we replaced *ecosystem* with *road density* at either 250 m and 1,000 m scales, gave similar results (Figures S5 and S6). We found a positive indirect effect of road density on red clover seed set mediated by CWM of body size and CWM of body size variation (for the 250 m scale; Sobel test; 2.231, *p < *.05; for the 1,000 m scale Sobel test; 2.000, *p < *.05; Figures S5 and S6).

## 4 DISCUSSION

We found three common and widespread bumblebee species to vary in their responses to urbanization. Foragers of *B. terrestris* were larger in cities, as expected due to urban fragmentation. However, this pattern was not observed for *B. lapidarius* and *B. pascuorum*, which did not differ in size between the two ecosystems as such. Interestingly though, at the 250 m scale, these two smaller bumblebees increased in size with road density, a major feature of urban fragmentation and a feature to which the body size of the larger *B. terrestris* responded at all scales. We also found, across ecosystems and for all three *Bombus* spp., higher ambient temperatures to be associated with a decrease in body size and an increase in variation in body size. At the community level, weighted mean body size and its variation increased with urbanization, with no concomitant change in abundance of the three species, while ambient temperature was negatively related with CWM body size and positively with CWM body size variation. Urbanization had an indirect, positive effect on the ecosystem service of pollination through its effects in boosting CWM body size and CWM body size variation.

Our results are based on a well‐replicated, statistically powerful, paired sampling design in nine independent central European cities and nine nearby rural sites, with a good numerical representation of all three bumblebee species for which we measured and compared body size. We measured many of the environmental correlates that might affect body size so as to explore potential drivers of change. We obtained results that were, overall, consistent with the predicted direction of change for both fragmentation and temperature (Merckx, Souffreau, et al., 2018). Nevertheless, we did not measure other variables that could potentially drive the observed differences, such as food (nectar and pollen) quality and plant community composition (Chole, Woodard, & Bloch, 2019; Quezada‐Euán et al., 2011). Overall, our results revealed that urbanization and ambient temperature have an impact on phenotypic shifts in bumblebee body size and on pollinator‐mediated plant reproductive success. We expand on these results below and place them in an eco‐evolutionary framework.

### 4.1 Population level response to temperature

Consistently for the three bumblebee species, ambient temperature at sampling was negatively related to body size. We did not, though, detect differences in ambient temperature between urban and rural sites at our points of sampling and thus the effects of ambient temperature on body size that we observed in bumblebees are unlikely to be linked to year‐long urban warming, which we have not quantified. The lack of a difference between ecosystems in ambient temperature when we collected bees could be due to our sampling from urban green spaces, which are cooler than nongreen spaces due to the evaporative and the shading effects of vegetation (Aram, Higueras García, Solgi, & Mansournia, 2019).

Nevertheless, ambient temperature varied across all of our sampling sites (rural and urban) and was a major predictor of bumblebee body size. Bumblebees are a group of large, well‐insulated, fury and cold‐adapted holometabolous insects. They actively control their nest temperature (Heinrich & Heinrich, 1983) using wing fanning and are able to regulate brood temperature at varied ambient temperatures (Kelemen & Dornhaus, 2018). Thus, while body size of workers is determined by nest temperature during larval growth and is therefore relatively independent of ambient air temperature, the performance of differently sized foragers is expected to be influenced by ambient air temperature; a larger forager should be able to maintain its thoracic temperature in colder temperatures whereas a smaller forager might be able to fly with reduced risk of overheating in warmer ambient temperatures (Goulson, 2010; Heinrich & Heinrich, 1983). These expectations, although reasonable, have been contradicted empirically; in an experimental study by Couvillon and Dornhaus (2009), large bumblebees (> 4.75 mm thorax width) successfully flew and foraged in high (36°C) ambient air temperatures. Furthermore, Peat, Darvill, Ellis, and Goulson (2005) found no evidence that ambient temperature affects the activity of *B. terrestris* workers of different body sizes. In our study, we sampled bumblebees in ambient temperatures ranging from 17°C to 28°C, which are well below the maximum thoracic temperature that bumblebees can tolerate (42–44°C; (Heinrich & Heinrich, 1983). Thus, even though large bumblebee workers do not like it warm (Heinrich & Heinrich, 1983), it seems unlikely that the observed negative relationship between body size and temperature which we detected is due to the effects of temperature per se on foraging activity of bumblebees of different sizes. We suggest that indirect effects of ambient temperature on worker behaviour, such as feeding rate in the field, food provisioning and larval feeding rate with knock‐on effects on larval nutrition and growth, might be more important. If, in warmer sites, workers allocate more time and energy in thermoregulating at the expense of larval feeding (Weidenmüller, 2004), successive cohorts of foragers might become smaller. Alternatively, or in addition, temperature might be correlated with the proliferation of warmth adapted parasites (Natsopoulou, McMahon, Doublet, Bryden, & Paxton, 2015), which might affect larval growth, or with competition for food resources from the warmth tolerant honeybees, known to affect negatively body size in bumblebees (Goulson & Sparrow, 2009).

Interestingly, ambient temperature was also positively correlated with variation (CV) in body size in all bumblebee species. This is in accord with the observation that size in a population tends to be more variable under stressful conditions (Molet, Péronnet, Couette, Canovas, & Doums, 2017; Tammaru & Teder, 2012). Though ours is a correlational study, similar results were also obtained in the experimental study of Kelemen and Dornhaus (2018) on *Bombus*, with high temperatures leading to increased variation in body size. Kelemen and Dornhaus (2018) attributed this, not to a direct effect of temperature on body size, but to an indirect effect of temperature on body size through larval feeding.

It is important to note that our discussion of the possible reasons for the observed relationships between temperature and body size (and its variation) relate to ambient temperature measured at sampling sites as bees were collected. For the same sites, we do not have the more detailed (across 24 hr, across the year) long‐term data that we would need to evaluate the possible wide‐ranging effects of urban warming on body size in bumblebees. However, if urban warming were the factor driving bumblebee body size, we would have expected smaller foragers in cities. That *B. terrestris* foragers were distinctively larger in cities suggests no dominant effect of urban warming shaping forager size, at least in *B. terrestris*.

### 4.2 Population level response to urban fragmentation

Urbanization had a direct influence on *B. terrestris* body size. The observed increase in body size of *B. terrestris* in cities is in line with the general shift observed in flying insects (Merckx, Souffreau, et al., 2018). Floral resources are known to affect body size as bumblebee adult body size is proportional to the amount of food received as a larva (Couvillon & Dornhaus, 2009). Given that we controlled for the availability of floral resources by sampling bees in flowering plant‐rich sites, the larger body size of *B. terrestris* observed in cities versus rural sites is likely due to the effects of urban fragmentation rather than to differences in the availability of resources. Indeed, roads, a major contributor to habitat fragmentation, showed a positive relationship with the mean body size of all three bumblebee species, while density of edges, where floral resources are usually located in fragmented landscapes, did not. We note, though, that flowering resources at the time of sampling might not reflect those at the time the sampled adults were being provisioned as larvae. Monitoring of floral resources across the life cycle of colonies would help address this question. However, if fragmentation acts by filtering for larger individuals that can fly longer distances whereas floral resources have developmental effects on body size (Couvillon & Dornhaus, 2009), then the flowers where a forager is sampled do not necessarily reflect her diet as a larva but her capacity of flying to them. Based on this reasoning, had our sampling not been restricted to flower‐rich sites, the body size shift we observed could have been even more pronounced. This is because, in the less accessible sites of the urban ecosystem, we would have sampled predominantly the larger individuals that managed to reach them.

The mean body size of our two other species, *B. lapidarius* and *B. pascuorum*, did not differ between urban and rural ecosystems as such, but, like *B. terrestris* did respond to road density, even though only at the 250 m scale. This result has two important implications: (a) given that road density is higher in urban sites, it confirms urbanization‐mediated fragmentation as the factor to which these bees respond across the urban/rural divide; (b) the scale at which our three bumblebee study species perceive the landscape and respond to environmental challenges is different. Their absolute size and foraging range differences are the most plausible explanations underlying the observed differences in intraspecific mean body size responses: *B. terrestris*, larger and with greater foraging range than *B. lapidarius* and *B. pascuorum*, might tend to fly beyond the mean patch size, while *B. lapidarius* and *B. pascuorum* are known to forage closer to their nests (Knight et al., 2005; Walther‐Hellwig & Frankl, 2000). Given that the latter two species are smaller, it is more likely that their energy requirements can be met locally, reducing their need to fly long distances to find food (Grab et al., 2019). It is noteworthy that even relatively small organisms like bee species, between which a difference in size might not seem so dramatic, still interact with the local environments at different scales. Moreover, they do so in accord with the hypothesis of a positive relationship between scale of effect and body size, in that small organisms are affected by environmental pressures at a smaller scale than large organisms (Thornton & Fletcher, 2014, but see Moll, Cepek, Lorch, Dennis, Robison, & Montgomery, 2020). This suggests that, also in bumblebees, body size correlates with the scale at which these organisms perceive their environment, probably because of the increased mobility of larger organisms.

That the average body size of *B. lapidarius* and *B. pascuorum* has increased significantly in the last 100 years in Belgium (Gérard et al., 2019) supports the idea of fragmentation as an environmental challenge, even for short‐range foragers such as *B. lapidarius* and *B. pascuorum*, and especially if coupled with depletion of food resources. We note that the study of Gérard et al. (2019) was not carried out along an urbanization gradient, but across a temporal increase in land‐use intensification of rural sites, which can be much more inhospitable for bees than cities (Hall et al., 2017; Samuelson, Gill, Brown, & Leadbeater, 2018; Theodorou et al., 2016, 2020). This could also be the reason why positive shifts in dispersal‐related traits have been found mainly in relation with land‐use intensification in bees (Gérard et al., 2019; Warzecha, Diekötter, Wolters, & Jauker, 2016), as in other taxa (Taylor & Merriam, 1995), rather than with increased urbanization. City life seems to elicit responses in body size that are taxon, context and scale dependent (Eggenberger et al., 2019; Merckx, Souffreau, et al., 2018; Piano et al., 2017). Additional studies are now needed to reconcile these differences in a wider conceptual framework that would allow to define common governing principles and more confident predictions about the direction of urbanization‐mediated trait changes.

Environmentally driven morphological changes arise as a result of two nonexclusive processes: selection and phenotypic plasticity (Grenier, Barre, & Litrico, 2016). In bees, body size is a highly plastic trait, with low heritability and major effects on fitness (Chole et al., 2019; Owen & McCorquodale, 1994). Within a bumblebee colony, workers, despite being highly related (*r* = .75), display as much as a 10‐fold difference in body size (Goulson, 2010), which is probably related to differential larval nutrition provided by the adult workers of the colony. Mobile organisms such as bees also have the opportunity to move through the landscape to find the environmental conditions that best match their phenotype, therefore maximizing their performance through habitat matching (Edelaar, Siepielski, & Clobert, 2008; Jacob, Bestion, Legrand, Clobert, & Cote, 2015). This could also underlie the body size distributions that we observe. More likely, habitat matching, local selection and plasticity act in concert (Edelaar et al., 2008), even though at different time scales. With our data, we cannot confirm or exclude any of these mechanisms. But that there was a consistent body size shift across three bee species is an indication that the inherent variability of bumblebee body size might allow a colony to adjust to the higher levels of fragmentation imposed by the urban setting, even though at different species‐specific scales. Given that the developmental control of body size in bumblebees is mainly nutritional (Couvillon & Dornhaus, 2009), and that bees are holometabolous insects, we do not argue for a direct developmental response of body size to different levels of fragmentation. Rather, we argue that bumblebee traits respond to the environment at the colony level; they are plastic in that a colony's work force is inherently variable in body size and a colony is therefore capable of phenotypic adjustments needed in a more fragmented environment. The question around the importance of plasticity in evolution is not new (Baldwin, 1896), and debate continues as to whether plasticity facilitates or hampers evolutionary change (Levis & Pfennig, 2020; Moczek et al., 2011; Pigliucci, Murren, & Schlichting, 2006; Robinson & Dukas, 1999; West‐Eberhard, 2003). Equally for bumblebees, the question of whether the body size shift we observed has already or will translate into evolutionary change (i.e. a change in allele frequency) is an open question that can be addressed only through carefully designed experiments (Edelaar et al., 2019) and concomitant genetic analyses (Schell, 2018; Theodorou et al., 2018).

### 4.3 Implications of community‐level body size shift for the ecosystem service of pollination

Changes in population body size distribution could alter ecological processes and could have major impacts on ecosystem services (Hendry, 2017; Merckx, Souffreau, et al., 2018; Rudman, Kreitzman, Chan, & Schluter, 2017) such as pollination, which is essential for plant reproduction and crop production (Klein et al., 2007; Ollerton, Winfree, & Tarrant, 2011; Woodcock et al., 2019). We detected a difference in body size distribution between rural and urban ecosystems, which was due to bumblebee individuals being overall larger in cities, rather than to an increase of the relative abundance of the larger *B. terrestris*. We confirmed bumblebees as being the dominant pollinators (Theodorou et al., 2016), which makes an understanding of their potential to respond to environmental change even more crucial, because of the presumably large negative impact on pollination their decrease would have. We also found that flower visitation rate was closely linked to pollination success, which is to be expected (Theodorou et al., 2020). Importantly, after controlling for visitation rates, urbanization also had a positive effect on pollination service provision via its direct effects on CWM body size and CWM variation in body size. Our results confirm body size as a predictor of pollination effectiveness (De Luca, Buchmann, Galen, Mason, & Vallejo‐Marín, 2019; Sahli & Conner, 2007; Willmer & Finlayson, 2014) and are important in that they explicitly link urbanization to a trait (body size) that affects a key ecosystem function (pollination), with potential repercussions at the ecosystem level. That not just community‐weighted mean body size (CWM) but also its variation (CWM CV) had positive effects on seed set of *T. pratense* underscores the importance of the variability of a trait, and not only its average, on the functioning of an ecosystem and confirms functional diversity as an important attribute of well‐functioning ecosystems (Petchey & Gaston, 2006; Thompson, Davies, & Gonzalez, 2015; Tilman et al., 1997). Body size‐related differences among individuals might lead to individual turnover in space and time, thereby increasing overall visitation to differentially accessible flowers in the inflorescence and facilitating complementarity in the use of floral resources among individuals and species (Woodcock et al., 2019), a mechanism linking trait variance to increased pollination service.

Phenotypic variation is both the result and the foundation of evolutionary and ecological processes (Darwin, 1859) and is critical for the maintenance of populations and for their short‐ and long‐term responses to novel and changing environments (Austin & Dunlap, 2019). It has implications for the sustained functioning of ecosystems (Hendry et al., 2011; Lankau, Jørgensen, Harris, & Sih, 2011). But only recently has the ecological importance and eco‐evolutionary implications of trait variance been re‐evaluated (Bolnick et al., 2011). Despite this renaissance of interest in variance, though, studies on the eco‐evolutionary implications of trait distributional change for pollination and its feedbacks on the community of pollinators are still lacking. We also need studies simultaneously considering multiple pressures on trait variances, particularly in urban settings where rapid trait changes are probably the result of multiple environmental challenges such as habitat loss and fragmentation, non‐native species, urban warming and environmental contaminants (Moll et al., 2019). All of these selective pressures might operate simultaneously (Alberti, Marzluff, & Hunt, 2017), but, not necessarily in the same direction (Merckx, Souffreau, et al., 2018). Moreover, they likely act on multiple traits, not just body size. Though we focused on body size in this study, the very complex nature of the urbanization process is very likely to affect other traits (Guenat, Kunin, Dougill, & Dallimer, 2019; Leonard, Wat, McArthur, & Hochuli, 2018). The combination of these pressures within urban areas provides a great opportunity to test and expand our current theories related to ecology, evolution and eco‐evolutionary dynamics (Alberti, 2015; Alberti, Marzluff, et al., 2017; Brans et al., 2017). Furthermore, a pressing problem is to feed a growing world population while reducing the human ecological footprint caused by agriculture (Godfray, 2010). Sustainable urban agriculture, which obviously would depend on urban pollination, might then become an important part of the solution (McDougall, Kristiansen, & Rader, 2019). Within this context, the potential benefits of urban environments for pollination, as shown in this and previous studies (Theodorou et al., 2016, 2017, 2020), deserve even more recognition. Additionally, appropriately designed studies would allow us to gain further insight in how to improve wild pollinator populations in urban areas, with positive implications for biodiversity and sustainable urbanization.

## CONFLICT OF INTEREST

The authors declare no competing interests with research described in this paper.

## AUTHOR CONTRIBUTION

AS and PT conceived the idea and contributed equally to the writing of the manuscript. PT participated in study design, collected and analysed the data. LB measured the bumblebee ITD. RJP participated in study design and data interpretation. All authors contributed in drafting and finalizing the manuscript.

## Supporting information

Supplementary Material

## Data Availability

Data are available in figshare. https://doi.org/10.6084/m9.figshare.12356372.v3
