# Supplementary material for: Urbanization is associated with shifts in bumblebee body size, with cascading effects on pollination
Source: Evol Appl. 2020 Aug 18;14(1):53–68. doi: 10.1111/eva.13087 (PMC7819558; doi:10.1111/eva.13087)
Supplement: Supplementary file 1 — Supplementary Material [file EVA-14-53-s001.docx]

**
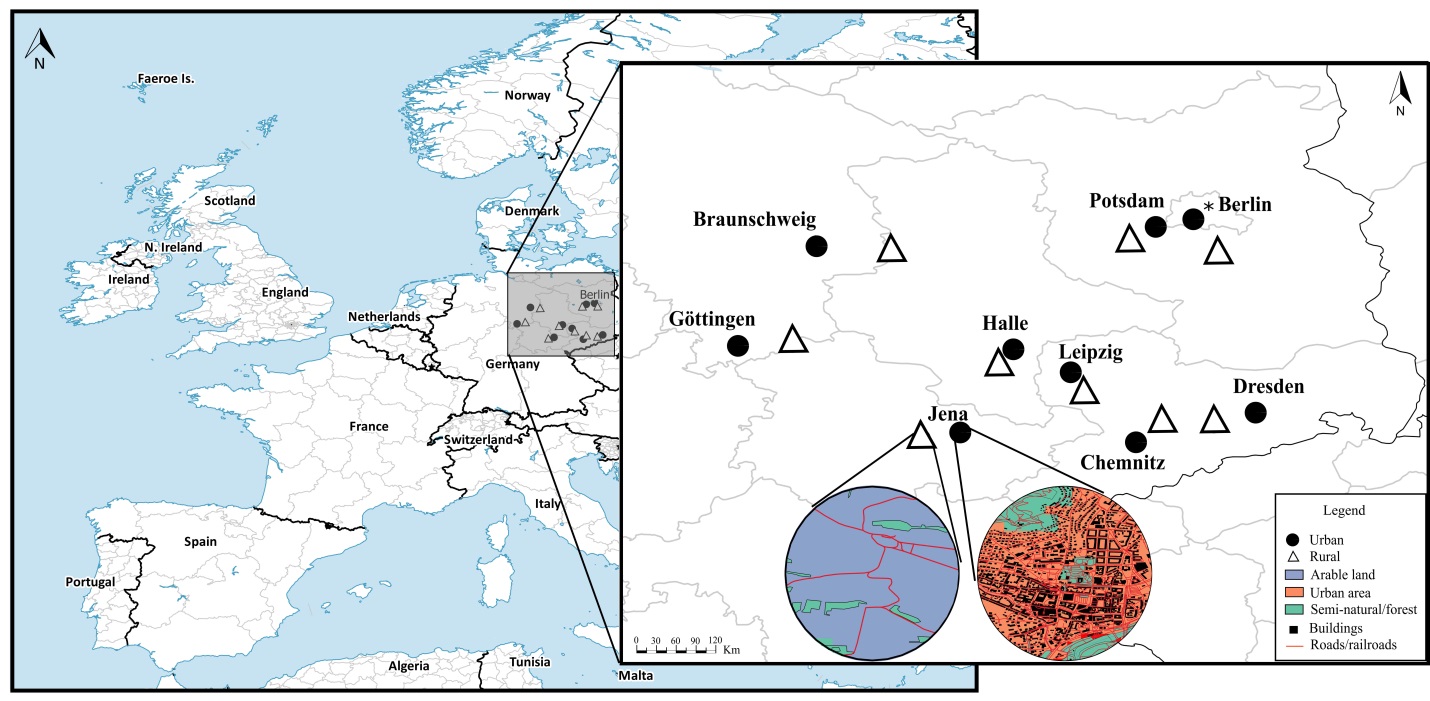
**

**Supplementary Figure 1.** Sampling sites in central and eastern Germany, where bumblebee workers were sampled. Rural sites are indicated with a white triangle while urban sites with a black circle. Highlighted are two sites (rural and urban), showing their landscape heterogeneity within a 1 km radius.


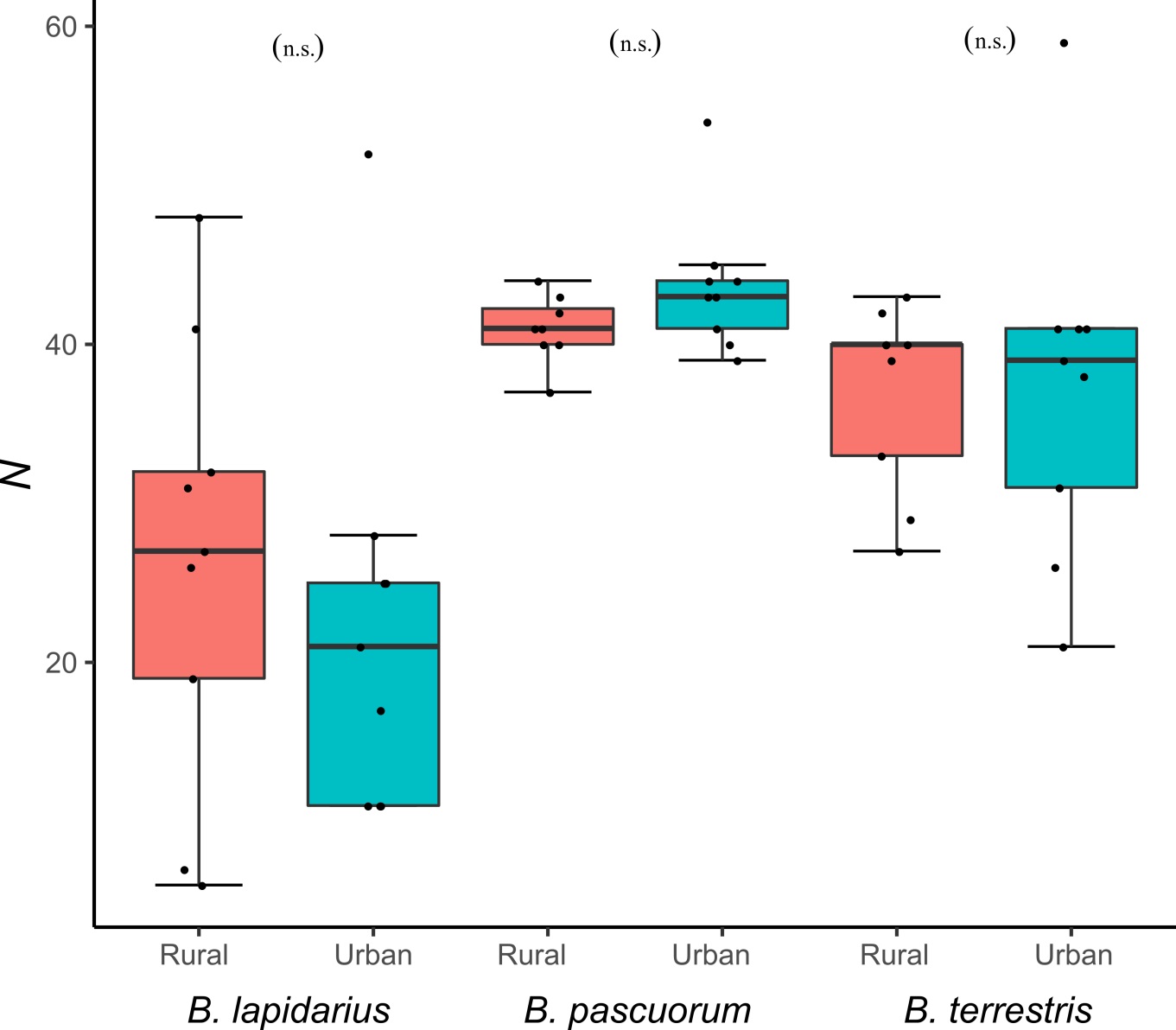


**Supplementary Figure 2**. Number of *Bombus lapidarius*, *Bombus pascuorum* and *Bombus terrestris* individuals collected and measured in rural and urban sites.


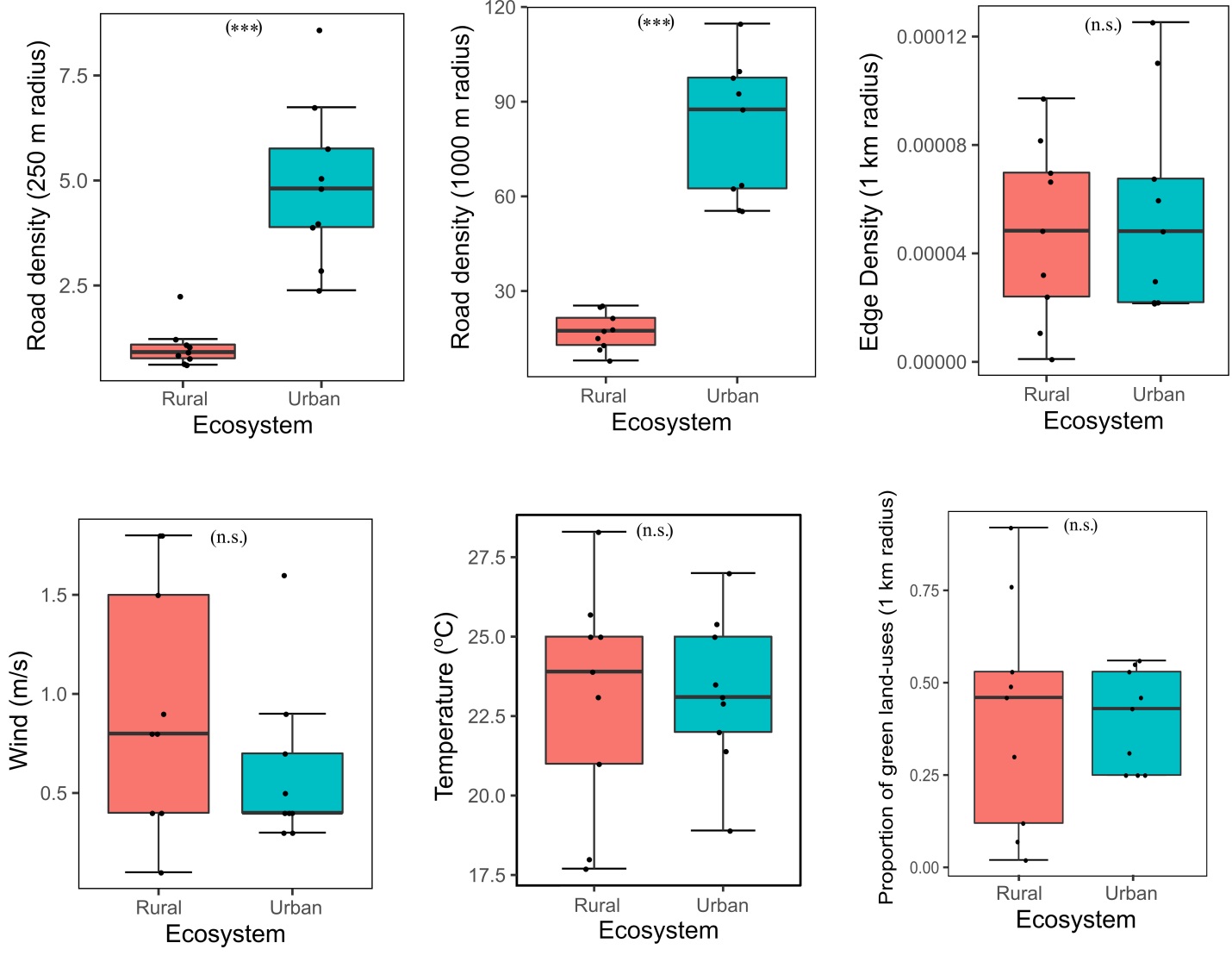


**Supplementary Figure 3.** Road density at the 250m and 1000m scales, edge density, wind speed, ambient temperature at the time of sampling and the proportion of green land-uses (including botanical and public parks, allotments, semi-natural and forest cover but excluding arable land) at the 1000 m scale, in rural *versus* urban sites. n.s. not significant; ***P≤0.001.


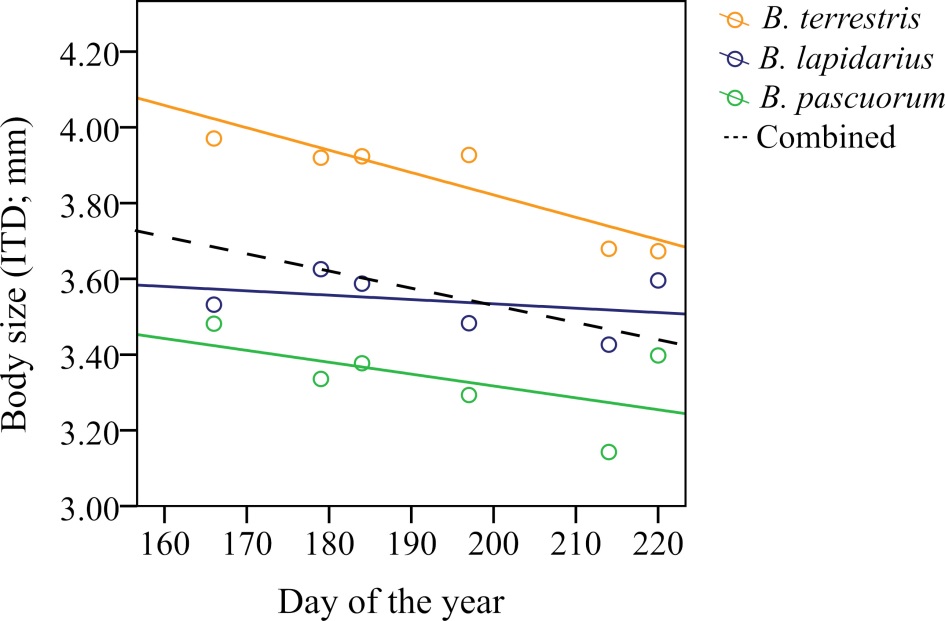


**Supplementary Figure 4**. Relationships between *Bombus* body size (ITD, intertegular distance) and day of the year. Plotted lines show predicted relationships.


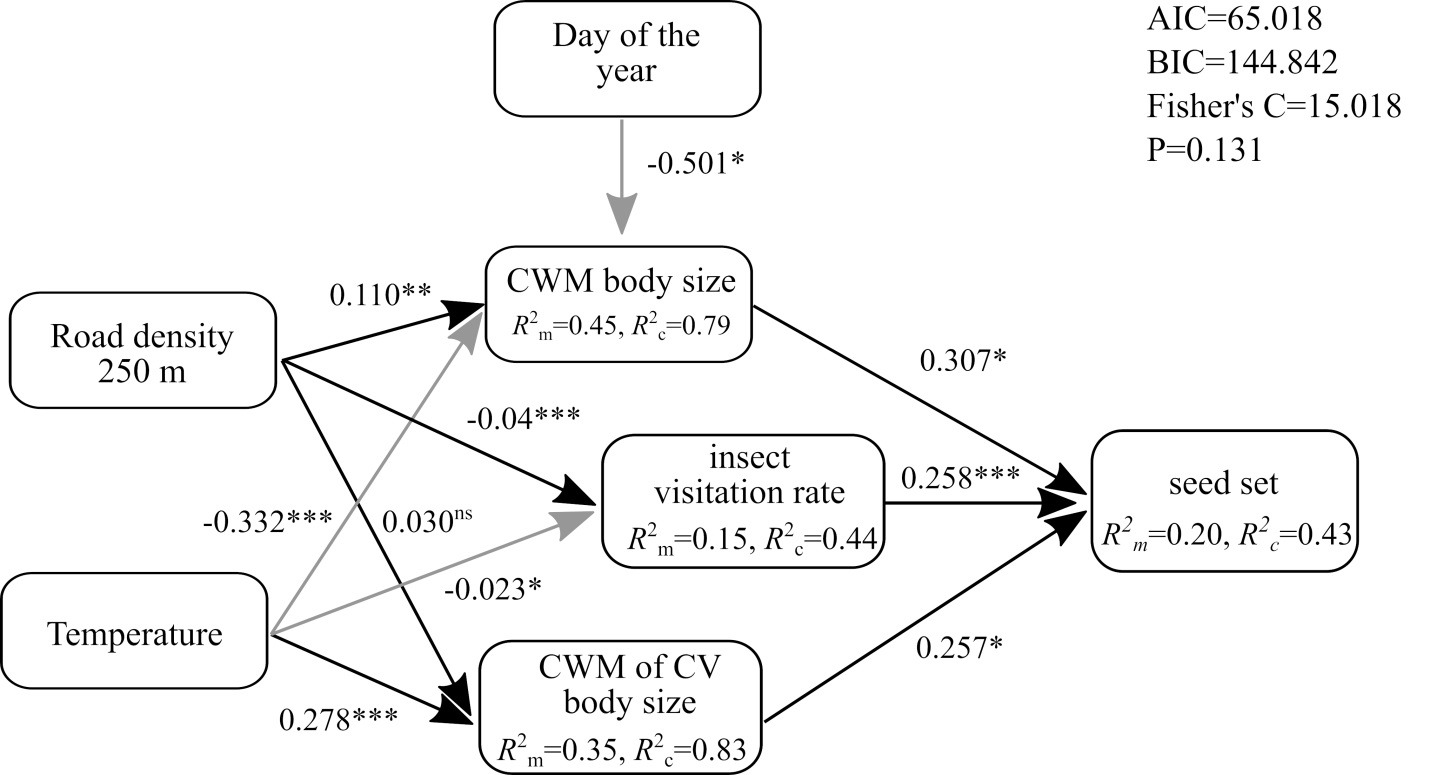


**Supplementary Figure 5**. Representation of the structural equation model of road density at the 250 m scale and ambient temperature, their relationships with bumblebee community-weighted mean (CWM) body size and CWM of the coefficient of variation (CV) of body size, and the effects of visitation rates and body size on pollination. Black solid arrows show positive and grey arrows negative effects, as derived from the piecewise SEM analysis. Standardized path coefficients are reported next to the bold arrows and *R ^2^* values (marginal *R^2^_m_* as well as conditional *R^2^_c_*) are reported for all response variables. ns not significant; **P*≤ 0.05; ***P*≤ 0.01; ****P* ≤ 0.001. In the top right of the figure we report the Akaike Information Criterion (AIC) and the Bayesian Information Criterion (BIC). Fisher’s C statistic was used for evaluating the fit of piecewise SEM. The statistic determines whether the model has a good fit (the model is not rejected when P > 0.05).


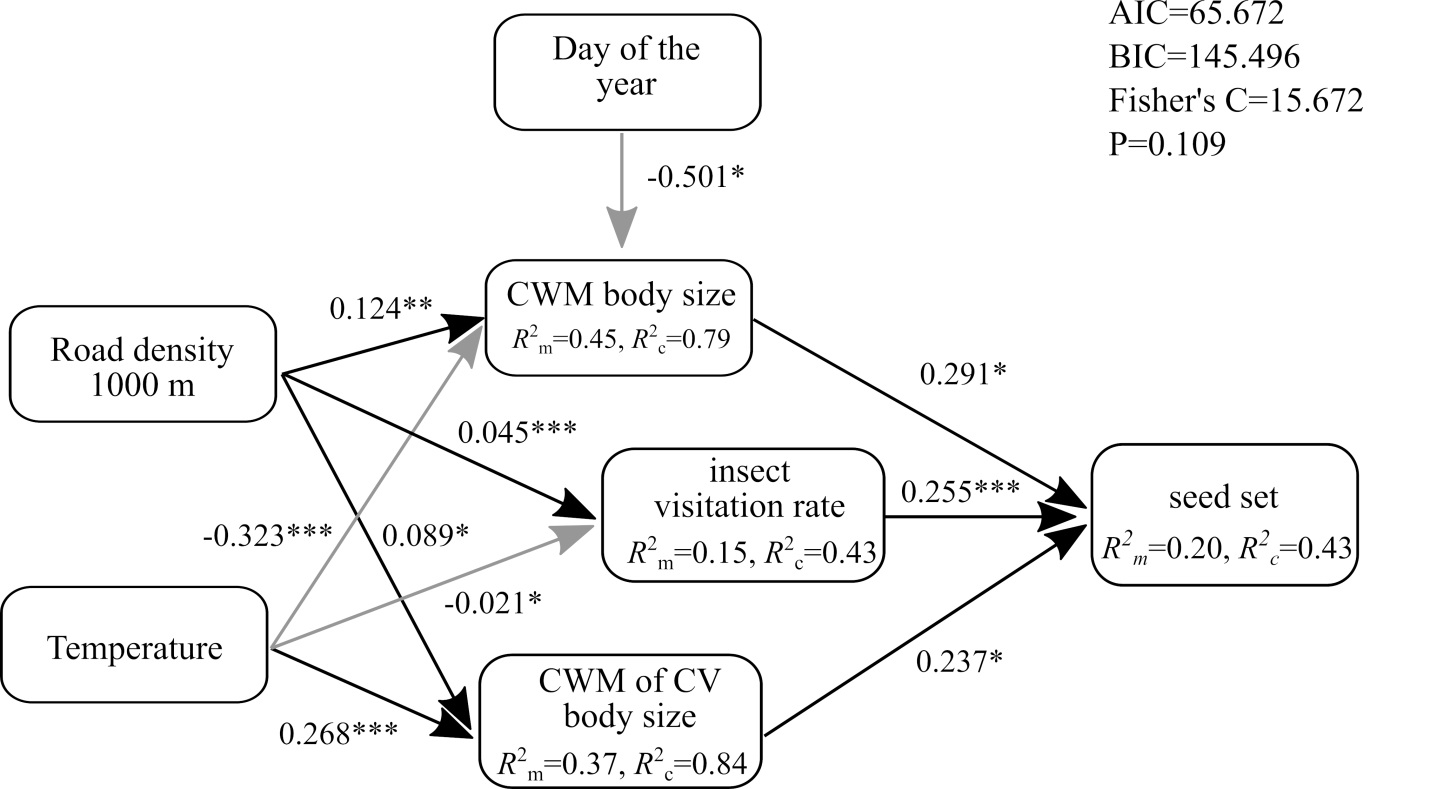


**Supplementary Figure 6.** Representation of the structural equation model of road density at the 1000 m scale and ambient temperature, their relationships with bumblebee community-weighted mean (CWM) body size and CWM of the coefficient of variation (CV) of body size, and the effects of visitation rates and body size on pollination. Black solid arrows show positive and grey arrows negative effects, as derived from the piecewise SEM analysis. Standardized path coefficients are reported next to the bold arrows and *R ^2^* values (marginal *R^2^_m_* as well as conditional *R^2^_c_*) are reported for all response variables. **P*≤ 0.05; ***P*≤ 0.01; ****P* ≤ 0.001. In the top right of the figure we report the Akaike Information Criterion (AIC) and the Bayesian Information Criterion (BIC). Fisher’s C statistic was used for evaluating the fit of piecewise SEM. The statistic determines whether the model has a good fit (the model is not rejected when P > 0.05).

| City |  | Proportion of each land cover class (arable, semi-natural, forest and four forms of urban land cover) at 1,000 m radius from a site centre | | | | | |  |  |  |
| --- | --- | --- | --- | --- | --- | --- | --- | --- | --- | --- |
|  | Arable | Semi-natural | Forest  (D=deciduous;;  M=Mixed) | Residential (domestic housing with gardens) | Commercial/  industrial | Botanical  park | Public  park | Allotments | Road density (km) | Building cover (km^2^) |
| Halle | 0 | 0 | 0.01 (D) | 0.63 | 0.10 | 0.20 | 0.04 | 0 | 55.40417 | 0.713536 |
| Leipzig | 0 | 0.15 | 0 | 0.52 | 0.02 | 0.14 | 0.12 | 0.05 | 92.65159 | 0.580860 |
| Jena | 0 | 0.02 | 0.04 (D) | 0.39 | 0.17 | 0.31 | 0.06 | 0 | 87.55788 | 0.716511 |
| Dresden | 0 | 0.08 | 0.18 (D) | 0.44 | 0.02 | 0.04 | 0.23 | 0 | 99.73007 | 0.312379 |
| Chemnitz | 0 | 0 | 0.20 (M) | 0.42 | 0 | 0.24 | 0.06 | 0.06 | 55.70457 | 0.328523 |
| Braunschweig | 0 | 0 | 0 | 0.64 | 0.05 | 0.25 | 0.06 | 0 | 97.63848 | 0.878783 |
| Potsdam | 0.04 | 0 | 0.22 (D) | 0.45 | 0 | 0.04 | 0.24 | 0.05 | 63.64427 | 0.150954 |
| Berlin | 0.03 | 0 | 0.01 (D) | 0.68 | 0.02 | 0.20 | 0.04 | 0 | 62.52504 | 0.561380 |
| Göttingen | 0 | 0.07 | 0 | 0.72 | 0.02 | 0.14 | 0.04 | 0 | 114.73180 | 0.946886 |
| Average | 0.01 | 0.03 | 0.07 | 0.54 | 0.04 | 0.17 | 0.10 | 0.02 | 78.73 | 0.58 |

**Supplementary Table 1.** Proportion of the main land cover classes at urban sites.

Green cover includes: both park types, allotments, semi-natural and forest cover

**Supplementary Table 2.** Proportion of the main land cover classes at rural sites; botanical and public parks were absent from all rural sites.

| Paired city name of rural site | Proportion of each land cover class (arable, semi-natural, forest and two forms of urban land cover) at 1,000 m radius from a site centre | | | | |  |  |  |
| --- | --- | --- | --- | --- | --- | --- | --- | --- |
|  | Arable | Semi-natural | Forest  (D=deciduous;  C=Coniferous)) | Residential  (domestic housing with gardens) | Commercial/  industrial | Allotments | Road density (km) | Buliding cover (km^2^) |
| Halle | 0.53 | 0.07 | 0.03 (D) | 0.34 | 0 | 0.02 | 25.39409 | 0.100467 |
| Leipzig | 0 | 0 | 0.76 (D) | 0.14 | 0.09 | 0 | 21.51763 | 0.055687 |
| Jena | 0.59 | 0.06 | 0.24 (D) | 0.10 | 0 | 0 | 7.99099 | 0 |
| Dresden | 0.42 | 0.04 | 0.49 (C) | 0.04 | 0 | 0 | 15.13525 | 0.018657 |
| Chemnitz | 0.62 | 0.05 | 0.02 (C) | 0.22 | 0.09 | 0 | 12.90439 | 0.034797 |
| Braunschweig | 0.52 | 0.02 | 0.44 (D) | 0.02 | 0 | 0 | 17.41563 | 0.004461 |
| Potsdam | 0.41 | 0 | 0.49 (C) | 0.07 | 0.01 | 0 | 25.05485 | 0.038421 |
| Berlin | 0 | 0.44 | 0.48 (C) | 0.01 | 0.05 | 0 | 17.86504 | 0.096161 |
| Göttingen | 0.95 | 0 | 0.02 (D) | 0.02 | 0 | 0 | 11.52529 | 0.011836 |
| Average | 0.45 | 0.09 | 0.33 | 0.10 | 0.02 | 0 | 17.20 | 0.04 |

Green cover includes: allotments, semi-natural and forest cover

**Supplementary Table 3.** Coordinates of field sites used in our study, sampling dates and weather during *Trifolium pratense* observations and sampling of bumblebees.

| Site | Latitude  (N) | Longitude  (E) | Sampling dates  (day/month) in 2014 | Weather  (morning, 0930-1000) | | |
| --- | --- | --- | --- | --- | --- | --- |
|  |  |  |  | Temperature (°C) |  | Wind (ms^-1^) |
| Rural Halle | 51.39112 | 11.87891 | 12/06-17/06 | 17.7 |  | 1.8 |
| Urban Halle | 51.48966 | 11.96135 | 12/06-17/06 | 25.4 |  | 0.9 |
| Rural Leipzig | 51.18594 | 12.49890 | 26/06-01/07 | 25.7 |  | 0.4 |
| Urban Leipzig | 51.32920 | 12.39198 | 26/06-01/07 | 18.9 |  | 0.4 |
| Rural Jena | 50.82824 | 11.30146 | 26/06-01/07 | 18.0 |  | 1.8 |
| Urban Jena | 50.93119 | 11.58430 | 26/06-01/07 | 21.4 |  | 0.5 |
| Rural Dresden | 50.94165 | 13.43837 | 02/07-06/07 | 23.9 |  | 1.5 |
| Urban Dresden | 51.04314 | 13.75754 | 02/07-06/07 | 22.9 |  | 0.7 |
| Rural Chemnitz | 50.96313 | 13.08918 | 02/07-06/07 | 28.3 |  | 0.9 |
| Urban Chemnitz | 50.85040 | 12.89103 | 02/07-06/07 | 23.5 |  | 0.4 |
| Rural Braunschweig | 52.20853 | 11.11153 | 15/07-19/07 | 25.0 |  | 0.4 |
| Urban Braunschweig | 52.26870 | 10.53336 | 15/07-19/07 | 23.1 |  | 0.3 |
| Rural Berlin | 52.16986 | 13.48448 | 31/07-03/08 | 25.6 |  | 0.8 |
| Urban Berlin | 52.45289 | 13.31002 | 31/07-03/08 | 25.0 |  | 1.6 |
| Rural Potsdam | 52.28192 | 12.83659 | 31/07-03/08 | 21.0 |  | 0.1 |
| Urban Potsdam | 52.40796 | 13.02213 | 31/07-03/08 | 27.0 |  | 0.3 |
| Rural Göttingen | 51.54377 | 10.38625 | 6/08-10/08 | 25.0 |  | 0.8 |
| Urban Göttingen | 51.53826 | 09.93850 | 6/08-10/08 | 22.0 |  | 0.4 |

#

# Supplementary Table 4. Total patch flower abundance in 10 x 1 m^2^ quadrats, and number of inflorescences of co-flowering *Trifolium pratense* plants within a 200 m buffer at urban and rural sites.

| Urban | Flower abundance | No. inflorescences of co-flowering *T. pratense* plants |
| --- | --- | --- |
| Halle | 87.4 | 3036 |
| Leipzig | 66.6 | 68 |
| Jena | 184.8 | 1600 |
| Dresden | 44.1 | 182 |
| Chemnitz | 84.3 | 426 |
| Braunschweig | 18.2 | 20 |
| Potsdam | 30.7 | 1020 |
| Berlin | 27.1 | 340 |
| Göttingen | 81.7 | 40 |
| Average | 77.2 ± 46.1 SD | 748 ± 1007 SD |
| Rural (paired to the like-named city) |  |  |
| Halle | 21.5 | 1869 |
| Leipzig | 150.8 | 376 |
| Jena | 58.9 | 1256 |
| Dresden | 93.7 | 400 |
| Chemnitz | 87.5 | 2100 |
| Braunschweig | 119.5 | 1400 |
| Potsdam | 27.1 | 55 |
| Berlin | 123.1 | 20 |
| Göttingen | 27.0 | 500 |
| Average | 73.7 ± 47.9 SD | 886 ± 784 SD |

# Supplementary Table 5. Pearson correlation coefficients (r) of the relationship between *Bombus* spp. body size (intertegular distance, ITD in mm) and road density at increasing area (given as radius in metres) from the centre of a site. The largest correlation coefficient is given in bold. All correlation coefficients are positive.

| Radius | 250 m | 500 m | 750 m | 1000 m |
| --- | --- | --- | --- | --- |
| *Bombus terrestris* ITD | 0.131 | 0.142 | 0.140 | **0.160** |
| *Bombus lapidarius* ITD | **0.026** | 0.006 | 0.000 | 0.001 |
| *Bombus pascuorum* ITD | **0.081** | 0.048 | 0.039 | 0.041 |

# Supplementary Table 6. Pearson correlation coefficients (r) of the relationship between *Bombus* spp. body size (intertegular distance, ITD in mm) and proportion of green cover (including botanical and public parks, allotments, semi-natural and forest cover but excluding arable land) at increasing area (given as radius in metres) from the centre of a site. The largest correlation coefficient is given in bold. All correlation coefficients are positive.

| Radius | 250 m | 500 m | 750 m | 1000 m |
| --- | --- | --- | --- | --- |
| *Bombus terrestris* ITD | 0.011 | 0.063 | 0.000 | **0.106** |
| *Bombus lapidarius* ITD | 0.091 | 0.102 | 0.091 | **0.133** |
| *Bombus pascuorum* ITD | 0.078 | 0.068 | 0.053 | **0.084** |

# Supplementary Table 7. Pearson correlation coefficients (r) of the relationship between *Bombus* spp. body size (intertegular distance, ITD in mm) and edge density at increasing area (given as radius in metres) from the centre of a site. The largest correlation coefficient is given in bold. All correlation coefficients are positive.

| Radius | 250 m | 500 m | 750 m | 1000 m |
| --- | --- | --- | --- | --- |
| *Bombus terrestris* ITD | 0.124 | 0.102 | 0.000 | **0.143** |
| *Bombus lapidarius* ITD | 0.106 | 0.107 | 0.002 | **0.136** |
| *Bombus pascuorum* ITD | 0.058 | 0.063 | 0.049 | **0.087** |

**Supplementary Table 8.** Best linear mixed effect models explaining body size (intertegular distance, ITD, mm), body size variation (coefficient of variation (CV) of ITD, %) and *Trifolium pratense* seed set across all our sampling sites. Model 1 and models 2 and 3 differ in that the predictor *road density* of models 2 and 3 replaces the predictor *ecosystem type* of model 1. Blap=*Bombus lapidarius*, Bpasc=*B. pascuorum*, Bter=*B. terrestris*, U=Urban, R=rural.

| **Response variable** | **Predictors** | **Estimate** | **SE** | **t-value** | **P-value** |
| --- | --- | --- | --- | --- | --- |
| Body size |  |  |  |  |  |
| (model 1) | Ambient temperature | -0.033 | 0.009 | -3.414 | <0.001*** |
|  | Day of the year | -0.070 | 0.026 | -2.655 | 0.032* |
|  | EcosystemR:SpeciesBlap | -0.381 | 0.030 | -12.696 | <0.001*** |
|  | EcosystemU:SpeciesBlap | -0.384 | 0.031 | -12.085 | <0.001*** |
|  | EcosystemR:SpeciesBpasc | -0.592 | 0.027 | -21.437 | <0.001*** |
|  | EcosystemU:SpeciesBpasc | -0.576 | 0.026 | -21.972 | <0.001*** |
|  | EcosystemR:SpeciesBter | -0.134 | 0.027 | -4.913 | <0.001*** |
|  |  |  |  |  |  |
| Body size | Ambient temperature | -0.030 | 0.010 | -3.003 | 0.002** |
| (model 2) | Day of the year | -0.075 | 0.025 | -2.953 | 0.020* |
|  | Species [T.Bpasc] | -0.205 | 0.021 | -9.429 | <0.001*** |
|  | Species [T.Bter] | 0.314 | 0.021 | 14.294 | <0.001*** |
|  | Road density (250m) | 0.027 | 0.009 | 3.009 | 0.002** |
|  |  |  |  |  |  |
| Body size | Ambient temperature | -0.051 | 0.011 | -4.290 | <0.001*** |
| (model 3) | Day of the year | -0.075 | 0.031 | -2.391 | 0.047* |
|  | Road density (1000m): SpeciesBlap | 0.018 | 0.020 | 0.861 | 0.389^ns^ |
|  | Road density (1000m): SpeciesBpasc | -0.024 | 0.016 | -1.507 | 0.132^ns^ |
|  | Road density (1000m): SpeciesBter | 0.057 | 0.016 | 3.560 | <0.001*** |
|  |  |  |  |  |  |
| CV body size | Species [T.Bpasc] | 0.011 | 0.006 | 1.815 | 0.076^ns^ |
| (model 4) | Species [T.Bter] | 0.015 | 0.005 | 2.798 | 0.007** |
|  | Ambient temperature | 0.006 | 0.002 | 3.220 | 0.002** |
|  | Sample size | -0.0003 | 0.002 | -0.113 | 0.910^ns^ |
|  |  |  |  |  |  |
| *T. pratense* seed set  (model 5) | Visitation rates | 1.731 | 0.339 | 5.095 | <0.001*** |
|  | CWM body size | 1.492 | 0.569 | 2.622 | 0.010* |
|  | CWM of CV body size | 1.412 | 0.549 | 2.574 | 0.011* |

ns non-significant, * P<0.05, ** P<0.01, *** P<0.001.

# Supplementary Table 9. Table of path coefficients from the piecewise SEM of the relationships between ecosystem, ambient temperature at sampling, day of the year, *Trifolium pratense* seed-set, *T. pratense* flower visitation rate and bumblebee body size across all 18 rural and urban ecosystems (visualization: Figure 3 in the main text). CWM = Community Weighted Mean; ITD = InterTegular Distance; CV = Coefficient of Variation

| Response |  | Predictor | Estimate (Standardized) | | | S.E. | | P |
| --- | --- | --- | --- | --- | --- | --- | --- | --- |
|  |  |  | |  |  | |  | |
| CWM ITD | 🡨 | Temperature | | -0.371 | 0.043 | | <0.001*** | |
| CWM ITD | 🡨 | Ecosystem (T. Urban) | | 0.303 | 0.070 | | <0.001*** | |
| CWM ITD | 🡨 | Day of the year | | -0.495 | 0.213 | | 0.053 ns | |
| CWM ITD CV | 🡨 | Temperature | | 0.253 | 0.046 | | <0.001*** | |
| CWM ITD CV | 🡨 | Ecosystem (T. Urban) | | 0.172 | 0.066 | | 0.010* | |
| Visitation rate | 🡨 | Ecosystem (T. Urban) | | 0.398 | 0.053 | | <0.001*** | |
| Visitation rate | 🡨 | Temperature | | -0.308 | 0.064 | | <0.001*** | |
| *T. pratense* seed set | 🡨 | CWM ITD | | 0.333 | 0.118 | | 0.005** | |
| *T. pratense* seed set | 🡨 | CWM ITD CV | | 0.268 | 0.114 | | 0.020* | |
| *T. pratense* seed set | 🡨 | Visitation rate | | 0.343 | 0.071 | | <0.001*** | |

S.E. = standard error; P = statistical significance; n.s. not significant; * P≤0.05; **P≤0.01; ***P≤0.001

**Supplementary Figure Model Assumptions**.

**Model 1 of Table 1**

**Model 2 of Table 1**

**Model 3 of Table 1**

**Model 4 of Table 1**

**Model 5 of Table 1**
